# Cognitive behavioral therapy for insomnia combined with eszopiclone for the treatment of sleep disorder patients transferred out of the intensive care unit: A single-centred retrospective observational study

**DOI:** 10.1097/MD.0000000000012383

**Published:** 2018-09-14

**Authors:** Ying Zhang, Jun Su, Jingquan Wang, Guangzhang Tang, Wei Hu, Jinghong Mao, Wanwen Ren, Yi Liu, Zhenghe Yu

**Affiliations:** a Department of Medical Psychosomatic, Hangzhou Seventh People's Hospital; b Department of Intensive Care Unit, Hangzhou First People's Hospital, Hangzhou; c Department of Intensive Care Unit , Anhui Provincial Hospital Affiliated to Anhui Medical University, Hefei, China.

**Keywords:** cognitive behavioral therapy for insomnia, eszopiclone, intensive care unit, sleep disorders

## Abstract

Patients transferred out of the intensive care unit (ICU) are always impaired by sleep disorders. Cognitive behavioral therapy for insomnia (CBT-I) and eszopiclone are 2 commonly prescribed strategies for insomnia. In the current study, the effect of the combined application of the 2 methods on sleep disorders in ICU transferred out patients was assessed.

Twenty-nine insomnia patients receiving combined treatment of CBT-I and eszopiclone and a corresponding number of patients treated with eszopiclone were collected. The incidence of discomfort experiences in ICU was recorded. Polysomnogram (PSG), Pittsburgh Sleep Quality Index (PSQI), self-rating anxiety scale (SAS), self-rating depression scale (SDS), and treatment emergent symptom scale (TESS) were used to assess the treatment efficacy and side effects.

Hospitalization for over 7 days, use of benzodiazepines, and experiencing anxiety, insomnia, and mechanical ventilation increased chances of sleep disorders. The sleep latency, awakening time, and total sleep time were further improved in patients treated with the combined therapy than patients treated with eszopiclone (*t* = −2.334, −2.412, 2.383, *P < *.05). Similar changing pattern was observed for PSQI score (*t* = −2.262, *P < *.05). The improvement effect of the combined therapy on the sleep efficacy, SWS phase III, and rapid eye movement sleep was also significantly stronger (*t =* 2.112, 2.268, 2.311, *P < *.05). Moreover, the SAS and SDS scores in patients treated with the combined therapy decreased more than those of patients treated with eszopiclone.

The efficacy of CBT-I combined with eszopiclone in the treatment of sleep disorders in ICU transferred out patients was better than eszopiclone.

## 1 Introduction

The number of patients transferred out of the intensive care unit (ICU) has kept increasing in recent years as a direct consequence of the rapid development of intensive care medicine.^[1]^ However, a large proportion of ICU transferred out patients are likely to be impaired by a series somatopsychic disturbances, such as ICU-acquired asthenia, cognitive disorders, or post-traumatic stress disorder (PTSD), which are combined defined as post-intensive care syndrome (PICS).^[1,2]^ It is well recognized that PICS is attributed to the diseases, complicated conditions in ICU, and restricted motion in ICU. Of different types of PICS, sleep disorders have been proved to be the most commonly diagnosed type and impact the recovery and normal life of ICU transferred out patients, which makes a great issue to caregivers.^[3]^

Currently, the major strategies to improve sleep conditions include nondrug therapy and drug therapy.^[4]^ Nondrug therapy can be further classified into psycho-behavior therapy and physiotherapy. Nevertheless, the application of nondrug therapy is restricted due to the poor compliance of the patients just transferred out of ICU.^[5]^ Regarding drug therapy, the most widely prescribed drugs in clinic are sedative-hypnotic drugs and antidepressants.^[4,6]^ Compared with nondrug therapy, the treatment efficacy of drug therapy is better based on clinic trails, but risk factors such as side effect and addiction of drugs also make the application of drug therapy rendered less satisfactory.^[7]^ Therefore, the current purpose for handling sleep disorders of patients transferred out of ICU is to improve sleep quality and to reduce side effects associated with different therapies. To fulfill the purpose, novel strategies with considerable treatment efficacy but mild side effects are demanding.

Cognitive behavioral therapy for insomnia (CBT-I) is a widely used method to improve sleep quality by correcting the incorrect cognition and bad habits of patients in sleep.^[8]^ The high treatment efficacy and long-term effect of the therapy have been validated in multiple clinic trials. For example, it is concluded that 70% to 80% of middle-aged adults with insomnia benefited from CBT-I.^[9–11]^ Moreover, for young- and middle-aged individuals, CBT-I improved sleep quality more compared with pharmacotherapy.^[12]^ Therefore, the method has been employed as the first-line method for the management of insomnia.^[8]^ Eszopiclone is a γ-aminobutyric acid (GABA)-receptor agonist whose safety and sustained efficacy for the treatment of insomnia have been previously demonstrated.^[13–15]^ The potential of eszopiclone to improve sleep maintenance without tolerance makes it a promising adjunctive agent for the treatment of insomnia. Although the treatment efficacy for insomnia of the 2 strategies has been well recognized, few studies have employed either method in the treatment of sleep orders of patients transferred out of ICU. Moreover, given that the combined application of different therapies is becoming a central subject in the treatment of insomnia.^[12,16]^ it was reasonable to explore the potentially synergistic interaction between the 2 strategies in treating sleep orders in patients transferred out of ICU.

Therefore, the major purpose of the current study was to assess the treatment effect of the combined application of the 2 methods in treating sleep disorders of ICU transferred out patients. We retrospectively analyzed the outcomes of 29 patients receiving the combined therapy admitted in Hangzhou Seventh People's Hospital. The treatment efficacy of the combined therapy was also compared with the treatment with eszopiclone only. The findings outlined in the current study showed that the combined therapy had a better treatment efficacy than the treatment using eszopiclone.

## 2 Materials and methods

### 2.1 Patients

Patients were selected from ICU admissions from January 2015 to December 2016 in Hangzhou Seventh People's Hospital. The inclusion criteria were as follow: hospitalized in ICU for over 72 hours; after transferred out of ICU, the patients were impaired by sleep disorders for over one month based on the criteria of diagnostic and statistical manual of mental disorders (DSM-5)^[17]^; received the treatment of CBT-I combined with eszopiclone for the improvement of sleep condition. Those who had sleep disorders induced by mental disorders, epilepsy, brain trauma, brain tumor, encephalitis, or cerebrovascular disease and those who received treatments of hypnotics and antipsychotics were excluded from the database. According to the inclusion and exclusion criteria, 29 cases possessing detailed clinicopathological information were included in the analysis. Moreover, an equal number of patients receiving the treatment of eszopiclone were used as the control group for assessing the treatment efficacy of CBT-I combined with eszopiclone method. An informed consent was signed by each patient and included in the document. The study was approved by the ethic committee of Hangzhou Seventh People's Hospital for relating screening, inspection, and data collection of the patients. All works were undertaken following the provisions of the Declaration of Helsinki.

### 2.2 Treating strategy

For CBT-I treatment, patients received 4 weekly sessions of the therapy. The treatments were performed face-to-face or on telephone. In the first face-to-face interview, the patients were informed of the whole procedure and methods employed during the treatment. The detail content of CBT-I was recorded in documents and kept by the patients along with sleep diaries. Then face-to-face treatments were performed at the end of each week again and on-telephone treatments were performed every week for 30 minutes. The treatment protocol contained psychoeducation, sleep hygiene education, restriction of time in bed, stimulus control, cognitive therapy, and relaxation techniques. For eszopiclone treatment, the drug dose started with 3 mg/day for the first 2 weeks and then decreased to 2 mg/day for the third and fourth weeks. The drug was taken by the patients one hour before sleep.

### 2.3 Assessment measures

The general clinicopathological information (including gender, age, height, weight, job, education background, and marriage) and sleep condition in ICU of patients were collected. The improvement in sleep condition of patients receiving different therapies was assessed by polysomnography (PSG) and Pittsburgh Sleep Quality Index (PSQI). Parameters in PSG included total sleep time, sleep latency, awakening time, sleep efficiency, and sleep structure. The emotion condition of patients was detected with self-rating anxiety scale (SAS) and self-rating depression scale (SDS). The side effect associated with the treatment was assessed by treatment emergent symptom scale (TESS). Different screenings were performed by a trained clinician blind to the study purpose and design.

### 2.4 Statistical analysis

Continuous data were represented as mean ± standard deviation (SD) and category data were represented as median with interquartile range (IQR). The differences in parameters between patients receiving different therapies were analyzed with student *t*-test or Chi-square text. All the tests were 2-tailed and a *P* value < .05 was determined to represent statistical significance. All statistical analyses were performed using SPSS 19.0.

## 3 Results

### 3.1 Participant information

Based on the inclusion and exclusion criteria, 29 patients were selected for the analysis of the treatment efficacy of the therapy using CBT-I combined with eszopiclone, including 14 males and 15 females. The average age and BMI for patients treated with the combined therapy were 54.6 ± 9.1 years old and 21.3 ± 2.4 kg/m^2^. A corresponding number of patients were selected from those who were treated with eszopiclone only. This control group included 15 males and 14 females, the average age and BMI of who were 46.1 ± 7.8 years old and 21.4 ± 4.1 kg/m^2^. Based on the statistical analyses, no significant difference was detected in gender, age and BMI between the 2 groups (*t =* 0.355, 0.041, 0.048, *P > *.05). The information regarding the use of analgesics and sedative, the uncomfortable experiences (pain and anxiety), hope caregivers, and memory of mechanical ventilation and invasive operation in ICU were also recorded and shown in Table 1.

**Table 1:**
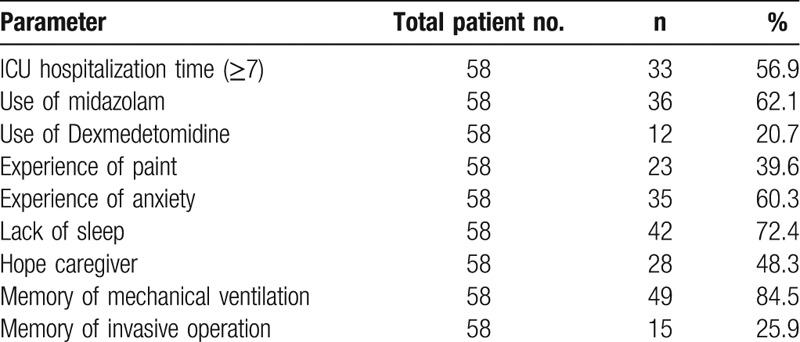
ICU hospitalization information.

### 3.2 Treatment efficacy of different therapies on PSQI score and PSG parameters

The treatment efficacies of different therapies were assessed by PSQI and PSG. The average PSQI scores before treatments were 17.57 ± 1.66 for patients treated with the combined therapy and 16.88 ± 3.76 for patients treated with eszopiclone, and no significant difference was detected between the 2 groups (Table 2). After the 4-week treatment, the average PSQI score was 6.63 ± 1.75 for patients treated with the combined therapy and 8.79 ± 2.48 for the eszopiclone therapy. It was found that both treatments significantly decreased the score of PSQI (*P < *.05) (Table 2). Moreover, statistically significant difference in PSQI score was also detected between the 2 groups after the treatments (Table 2), which was indicative of the higher treatment efficacy of the combined therapy against sleep disorders when compared with the eszopiclone therapy. Regarding the detection with using PSG, the changing patterns in total sleep time, sleep latency, awakening time, and sleep efficiency were in identical pattern to that of PSQI score (Table 2). For the sleep structure, the treatment efficacy of the combined therapy on slow-wave sleep (SWS) phases and rapid eye movement (REM) sleep was stronger than that of the eszopiclone therapy (Table 2).

**Table 2:**
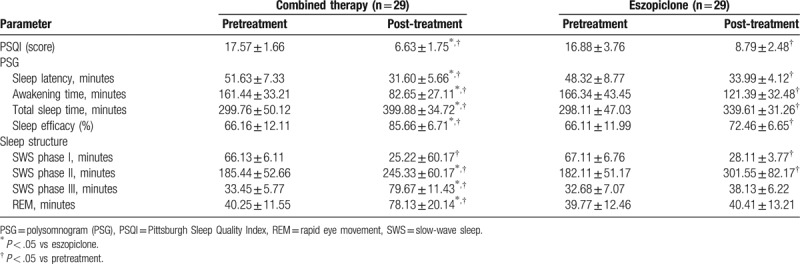
Treating efficacy of different therapies on PSQI score and PSG parameters.

### 3.3 Treatment efficacy of different therapies on SAS and SDS scores

No significant difference was detected between the 2 groups in SAS and SDS scores before the treatments (Table 3). After the 4-week treatments, both therapies significantly reduced the scores of SAS and SDS (Table 3). Moreover, the effect of the combined therapy on both scales was significantly stronger than that of the eszopiclone therapy, indicating that the combined therapy was more effective to attenuate emotion symptoms associated with sleep orders.

**Table 3:**
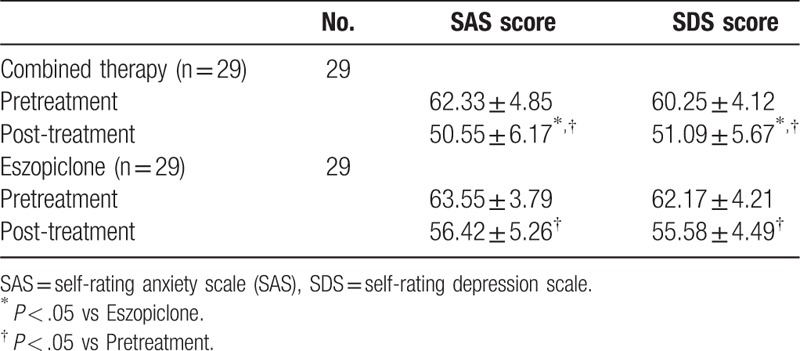
Treating efficacy of different therapies on SAS and SDS scores.

### 3.4 Assessment of the side effects associated with the 2 therapies

The side effects associated with the 2 therapies were assessed using TESS. For patients treated with the combined therapy, headache and nausea were recorded 2 times respectively, and dyspepsia and parageusia were recorded one time, respectively. For patients treated with the eszopiclone therapy, somnolence was recorded 2 times, and xerostomia and parageusia were recorded 2 times respectively. The side effects were mild and disappeared for no more than 3 days. No special management was employed for patients with side effects. Based on the assessment using TESS, no significant difference was detected between the 2 groups.

## 4 Discussion

Hospitalization in ICU is a tough experience for most patients, which will result in severe mental and physical abnormalities even after a long-term transferred out of ICU.^[18]^ The black mood, irritability, and anxiety induced by the confused time and restricted motion in ICU are together classified as PICS.^[18]^ The awful mental conditions of ICU transferred out patients directly influence their sleep quality: most of these patients can only have sleeps of lucid interval, SWS phase I and II while only few patients can experience sleeps of SWS phase III and REM. The poor sleep condition is always associated with the impaired functions of neurons in cerebral cortex, which further affects the mental and physical conditions of the patients.^[19]^ Based on our previous clinical investigations, for patients staying in ICU for more than 7 days, receiving benzodiazepine treatments, mechanical ventilation, or experiencing insomnia during the stay in ICU, a higher chance to be impaired by sleep disorders after their transferred-out was observed (unpublished data). Worse still, even if some strategies such as psychotherapy, physical therapy, or drugs are employed in attempt to attenuate the pain or anxiety associated with the ICU stay, more than 50% patients transferred out of ICU still keep the memory of pains induced by mechanical ventilation and invasive operation, and are more easily to develop sleep disorders.^[20]^ Therefore, the development of effective and mild treatment strategies for improving the sleep conditions of ICU transferred out patients is demanding prompt solution.

In the current study, we retrospectively compared the treatment efficacy of CBT-I combined with eszopiclone in improving the sleep conditions of patients transferred out of ICU. It is well recognized that more than 55% ICU transferred out patients will develop PTSD which is characterized by anxiety, depression, and sleep disorders.^[21]^ The improvement effect of CTB-I on PTSD has been long proved,^[22]^ especially for ameliorating insomnia associated with PTSD.^[23]^ By correcting the incorrect cognition and bad habits of patients in sleep, CBT-I effectively improves the sleep quality and has been proposed as the first-line therapy for the treatment of insomnia by WHO.^[24]^

Based on our analyses, the treatment of CBT-I combined with eszopiclone dramatically improved the sleep structure of patients transferred out of ICU, which was represented by the higher proportion of SWS phase III and REM. Compared with the therapy with eszopiclone only, the combined therapy had a solidly higher effect on SWS phase III and REM. It is reported that the function of eszopiclone is similar to that of benzodiazepines, which can effectively shorten the sleep latency, reduce awakening time, and increase total sleep time.^[25]^ However, neither type of the 2 drugs has obvious improvement effect on the proportion of SWS phase III and REM.^[25]^ Thus, our results were in consistence with the previous report. The increased proportion of SWS phase III can benefit patients by increasing the secretion of hormone involved in the growth and development and strengthening the immunocompetence of human bodies. Moreover, the increased the proportion of REM is also critical in the recovery of patients from impairments associated with ICU stay, which can increase the synthesis of proteins in brain tissues and retard aging of brain cells.^[26]^

Given that sleep disorders are not only physical diseases but also a process of psycho-disturbance, the effect of both therapies on the emotion states of patients was also analyzed in the current study. Compared with those treated with eszopiclone only, the SAS and SDS scores in patients treated with the combined therapy were significantly lower, further indicating that CBT-I had a synergistic effect with eszopiclone. The major treatment modalities of CBT-I include cognitive therapy, behavioral therapy, and sleep hygiene education, which can not only improve the sleep quality but also attenuate the psycho-disturbance of the patients.^[27,28]^ Taken the information above together, the combined therapy is characterized by the advantages of CBT-I and eszopiclone: CBT-I increases patients’ confidence in sleep and attenuates anxiety and depression symptoms of patients, and eszopiclone is effective in reducing sleep latency and increasing sleep efficiency. Although the considerable treatment effect of CBT-I on ICU-acquired sleep disorders has been previously demonstrated, the application of the strategy is being restricted by diverse factors such as the lack of operators, high cost, and long treatment time. Therefore, the combined application of CBT-I and eszopiclone that may lower the cost and shorten the treatment time can be served as an alternative option for traditional CBT-I. However, the results of the current study might be rendered less valuable due to the small sample size and the lack of long-term follow-up.

In conclusion, the findings outlined in the current study inferred that the combined application of CBT-I and eszopiclone was more effective in attenuating sleep disorders than the treatment of eszopiclone only. The sleep structure, efficiency, and the emotion state of the patients receiving the combined therapy were restored without significant side effects. To promote the application of the combined therapy, studies with larger sample size and long-term follow-up are needed in the future.

## Author contributions

**Conceptualization:** Jun Su.

**Data curation:** Ying Zhang, Jingquan Wang, Guangzhang Tang, Wei Hu, Wanwen Ren.

**Resources:** Guangzhang Tang.

**Software:** Jinghong Mao.

**Writing – original draft:** Ying Zhang, Jingquan Wang, Wei Hu.

**Writing – review & editing:** Jun Su, Yi Liu, Zhenghe Yu.
